# A New Strategy for the High-Throughput Characterization of Materials’ Mechanical Homogeneity Based on the Effect of Isostatic Pressing on Surface Microstrain

**DOI:** 10.3390/ma17030669

**Published:** 2024-01-30

**Authors:** Zhigang Fang, Qun Ren, Haizhou Wang, Jingyi Cao, Xuejing Shen, Wenyu Zhang, Weihao Wan, Wenchang Yin, Liang Li, Bolin Zang

**Affiliations:** 1 Naval Research Institute, People’s Liberation Army of China, Beijing 100072, China; 2 Beijing Advanced Innovation Center for Materials Genome Engineering, Central Iron & Steel Research Institute, Beijing 100081, China; 3 Beijing Key Laboratory of Metal Materials Characterization, NCS Testing Technology Co., Ltd., Beijing 100081, China

**Keywords:** high-throughput characterization, mechanical property, isostatic pressing, microstrain, heat-resistant steel

## Abstract

A new strategy for the high-throughput characterization of the mechanical homogeneity of metallurgical materials is proposed. Based on the principle of hydrostatic transmission and the synergistic analysis of the composition, microstructure, defects, and surface profile of the chosen material, the microstrain characteristics and changes in surface roughness after isostatic pressing were analyzed. After isostatic pressing, two types of microstrains were produced: low microstrain (surface smoothening with decreasing roughness) and large microstrain (surface roughening with increasing roughness). Furthermore, the roughness of the roughened microregions could be further classified based on the strain degree. The phenomenon of weak-interface damage with a large microstrain (plastic deformation, cleavage fracture, and tearing near nonmetallic inclusions) indicated that the surface microstrain analysis could be a new method of high-throughput characterization for microregions with relatively poor micromechanical properties. In general, the effect of isostatic pressing on the surface microstrain of heat-resistant steel provides a promising strategy for achieving high-throughput screening and statistically characterizing microregions with poor micromechanical properties, such as microregions containing microcracks, nonmetallic inclusions, pores, and other surface defects.

## 1. Introduction

Structural metallic materials are widely used in many fields, such as the aerospace, nuclear, weapon, military equipment, and petrochemical industries. The failure of structural metallic materials during service may lead to severe safety accidents, which could impact personnel safety and lead to economic losses and related irreversible damage [1,2,3]. During the service of metallic structural components, failure occurs first in regions with relatively poor mechanical properties and may then cause the entire failure of the components. For example, defects such as crack initiations that form near non-metallic inclusions could cause the failure of structural materials and may pose a safety risk [4,5]. Therefore, it is essential to find possible microregions with relatively poor mechanical properties, such as microregions containing non-metallic inclusions, pores, and other defects, in order to study material reliability and service safety as well as to develop a new technology for the high-throughput characterization of the micromechanical properties of materials [1,6].

The main types of mechanical testing approaches can be classified into macromechanical and micromechanical testing techniques. Macromechanical testing includes different types of techniques, such as compression, tension, torsion, bending, and fatigue testing [7,8,9,10]. Micromechanical testing includes several techniques, such as instrumental indentation methods (Vickers indentation and nanoindentation), micropillar compression, microtension, and microfatigue testing [11,12,13,14]. During the service process of structural metallurgical materials, component failure would first to occur in microregions or microareas with poor mechanical properties such as those with defects, inclusions, and pores in any possible area of the component [15]. For the entire screening of microregions with poor micromechanical properties, such as areas with nonmetallic inclusions, microcracks, and shrinkage porosity, developing new methods for achieving high-throughput characterization of the micromechanical properties of materials is necessary [15,16]. However, it is difficult to achieve the continuous cross-scale characterization of the micromechanical properties across the entire surface of specimens using the newly developed techniques, which are based on the principle of combinatorial technologies, such as the compression testing of micropillar arrays and the array testing of nanoindentation [11,16].

Typically, the isostatic pressing technique has been widely used in the fields of material densification and forming during casting, sintering, joining, and welding [17,18,19]. The isostatic pressing technique includes cold isostatic pressing (CIP), medium isostatic pressing (MIP), and hot isostatic pressing (HIP), characterized by different processing temperatures. For example, the evolution of the grain boundary network of 316L austenitic steel during densification was investigated using an HIP thermomechanical process [20]. Ceramic components with a complex shape can be manufactured through the combination of the CIP of selective laser-processed (SLP) parts and solid-state sintering (SSS) [21]. Owing to the principle of hydrostatic transmission, an equivalent load per unit area is applied to the surface, and a corresponding loading-induced strain is simultaneously generated [16]. In addition, due to the intrinsic inhomogeneities of the composition, microstructure, and mechanical properties, the strain exhibits different characteristics, which may provide a new method for the high-throughput screening and characterization of the micromechanical properties of materials [22].

In the present work, G115 heat-resistant steel was selected as a demonstration material, and the CIP technique was used to generate strain (by applying a load) on the specimen surface. By investigating the composition, microstructure, defects, and surface profile of this material, the homogeneity of its micromechanical properties could be preliminarily examined by high-throughput screening and statistically analyzed.

## 2. Experimental Procedure

To analyze the effect of isostatic pressing on the surface microstrain and evaluate the application potential of this technique in the field of high-throughput mechanical screening and characterization, heat-resistant steel was used as an experimental material. This method requires 5 steps: (1) grind and polish the surface of the sample until no obvious scratches can be detected under an optical microscope on the surface of the sample; (2) use a microhardness or nanoindentation instrument to mark an area on the surface of the sample; (3) characterize the element content, microstructure, microdefects, and three-dimensional surface morphology of the sample before cold isostatic pressing in the test area based on the position coordinates of the test area; (4) perform cold isostatic pressing for strain processing on the polished surface and characterize element content, microstructure, microdefects, and three-dimensional surface morphology of the entire surface area of the metal sample after CIP treatment; (5) compare the composition, microstructure, microdefects, and three-dimensional surface changes of the sample before and after CIP and use software to statistically analyze the sample roughness to obtain a micromechanical evaluation with statistical characterization in a full field of view and cross-scale and high-throughput screening. In this work, all the apparatuses were calibrated by professional metrology calibration institutions. Microhardness was tested using an automatic microhardness tester (Qness 150A+, Wien, Austria) with a loading of 10 N and a holding time of 10 s. Moreover, the coordinate position could be established through indentation using the automatic microhardness tester. The specimens were polished using a metallographic polishing machine to obtain high-quality surfaces. The samples were processed via CIP with a pressure of 190 MPa and a holding time of 30 min. Energy-dispersive spectroscopy (EDS, Oxford, UK), micro X-ray fluorescence spectrometry (XRF, Bruker, Germany), automatic optical microscopy (OM), high-throughput field-emission scanning electron microscopy (high-throughput SEM, FBT-NCS, Shanghai, China), tungsten filament scanning electron microscopy, and contour OM were utilized to study the composition, microstructure, defects, and surface profile of the samples. For the quantitative analysis of roughness, 100 independent rectangular areas of 100–300 × 100–300 μm^2^ were chosen for *S_a_* and *S_q_* statistics. The value of length × width was fixed, which provided good comparability of the result before and after CIP. The samples were processed by Gaussian filtering in short-wave passing mode.

## 3. Results and Discussion

**Figure 1:**
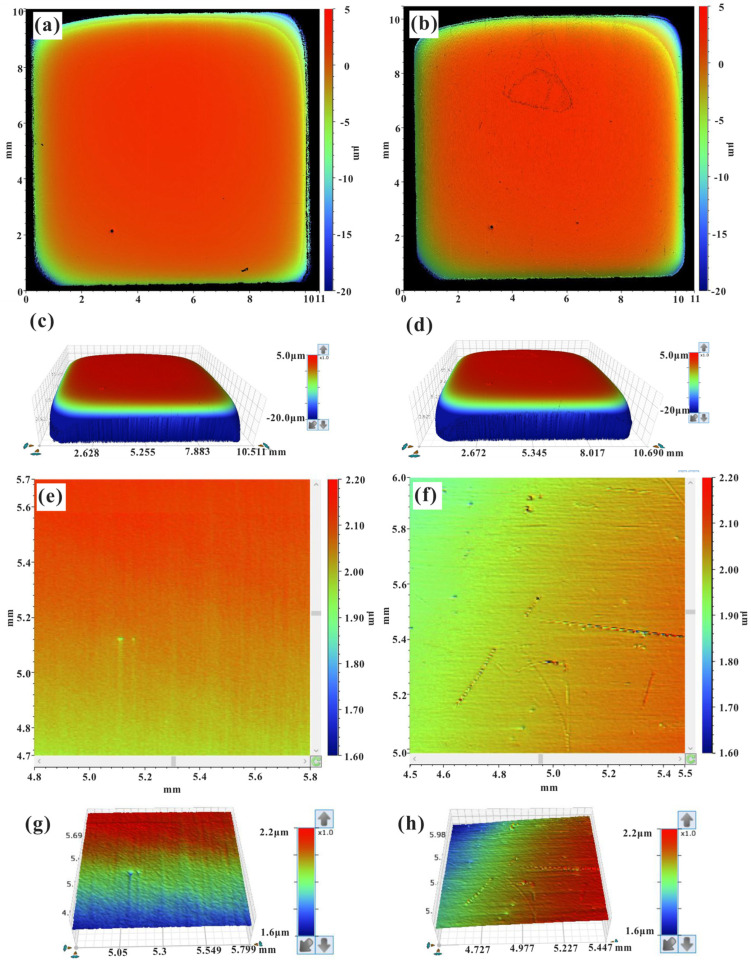
Surface profile of the heat-resistant steel specimen before and after CIP: (**a**) 2D contour map before CIP; (**b**) 2D contour map after CIP; (**c**) 3D profile of (**a**); (**d**) 3D profile of (**b**); (**e**) magnification of (**a**), (**f**) magnification of (**b**), (**g**) 3D profile of (**e**); (**h**) 3D profile of (**f**).

Figure 1 shows the two-dimensional (2D) contour map (Figure 1a,b,e,f) and three-dimensional (3D) (Figure 1c,d,g,h) surface profile data before and after CIP obtained via contour OM. The experimental results revealed a smooth surface of the heat-resistant steel before isostatic pressing, which was due to the excellent surface quality after metallographic polishing (see Figure 1a). In this study, we referred to the grinding and polishing requirements of conventional metallographic samples, which are met when the surface of a sample presents no significant damage or scratches. Therefore, the smoothness (roughness at the nanoscale level) referred to here was relative to the strain generated in the experiment, since the roughness after microstrain generation through CIP ranged from the submicron to the micron level. Significant variations in the local surface height were observed in a very small number of microregions (which were related to surface microstrains in μm scale, since the microstrain along the direction of the sample surface induced by CIP generally ranged from the submicron level to one or hundreds of micrometers, causing discontinuity in the surface contour. Moreover, microregions were defined also based on the resolution of the white light profilometer (in lateral direction but not in vertical direction). By contrast, numerous microregions with significant fluctuations in surface height could be detected on the heat-resistant steel surface obtained after isostatic pressing (Figure 1b). The magnified views of local areas in Figure 1a,b are also presented in Figure 1e,f, respectively. As observed for the whole surface area, regions exhibiting significant microstrain inhomogeneity appeared; these regions were characterized by different strain degrees.

As is well known from the deformation and shrinkage behavior of powder materials during isostatic pressing, a macroscopic shape variation results in inhomogeneous strain due to the densification tendency of powders [1,2,3]. Therefore, the strain at the center of the specimen is larger than at the edges, but the specimen surface in the corners becomes sharp after isostatic pressing [23,24]. For example, on the basis of the Drucker–Prager–Cap model, the densification and deformation of selective laser-sintered metal parts during isostatic pressing was reported by Du et al. [25]. In this previous work, it was found that the shear stress was small due to the uniform load applied to the specimen surface, and only a small shear strain was generated at the corners of the specimen, which led to sharpening at the edges of the specimen. Therefore, a surface profile may simultaneously contain macroscopic and microscopic information, and the surface in the corners of a specimen may exhibit a larger degree of distortion and deformation, induced by macroscopic shape variation. Thus, the ability to distinguish these two types of surface profile data is required. This will be discussed below in detail [24].

**Figure 2:**
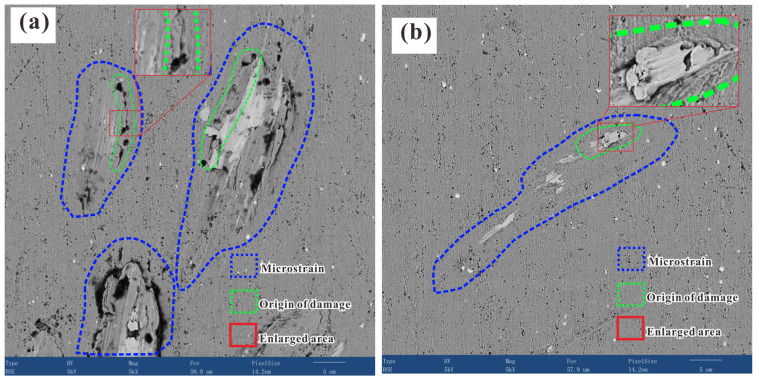
SEM images of the surface microstructures defects, and microstrain pattern of the heat-resistant steel specimen after isostatic pressing: (**a**) typical surface microstrains near non-metallic inclusions or pores; (**b**) typical surface microstrains near non-metallic inclusions.

When a load is applied uniformly to a liquid medium, hydrostatic transmission will occur through the liquid, and the load will then be uniformly applied to the specimen surface [26]. Similar to the testing of micromechanical properties, such as the evaluation of the microhardness of materials through the extrusion deformation of a specimen surface induced by indentation, micromechanical properties (such as hardness, strength, and residual stress) can be determined indirectly through the analysis of the relationship between load, loading time, and deformation behavior of materials during isostatic pressing because of the similarity of this test with that based on the extrusion principle [27,28]. Therefore, in order to analyze the micromechanical properties of heat-resistant steel in different microregions on the basis of the surface deformation induced by isostatic pressing, the deformation behavior should be analyzed and classified before analyzing the deformation mechanism [29]. Figure 2 shows the microstructure and morphology of the areas subjected to microstrain obtained via high-throughput SEM; two imaging modes were adopted, namely, the secondary electron (SE) and the backscattered electron (BSE) modes. From the analysis of the thousands of SEM images obtained via high-throughput SEM, it was found that the surface of heat-resistant steel after isostatic pressing was mainly subjected to two types of microstrain: low microstrain (mode I, surface smoothening with decreasing roughness, which will be discussed later) and large microstrain (mode II, surface roughening with increasing roughness depending on the degree of the microstrain). According to the degree of the microstrain, mode II could also be classified into two types, namely, mode II-A (microstrain at the submicron level) and mode II-B (microstrain at the micron level). Various types of surface strains caused by different individual deformation mechanisms or multiple deformation mechanisms could be detected between these two types.

**Figure 3:**
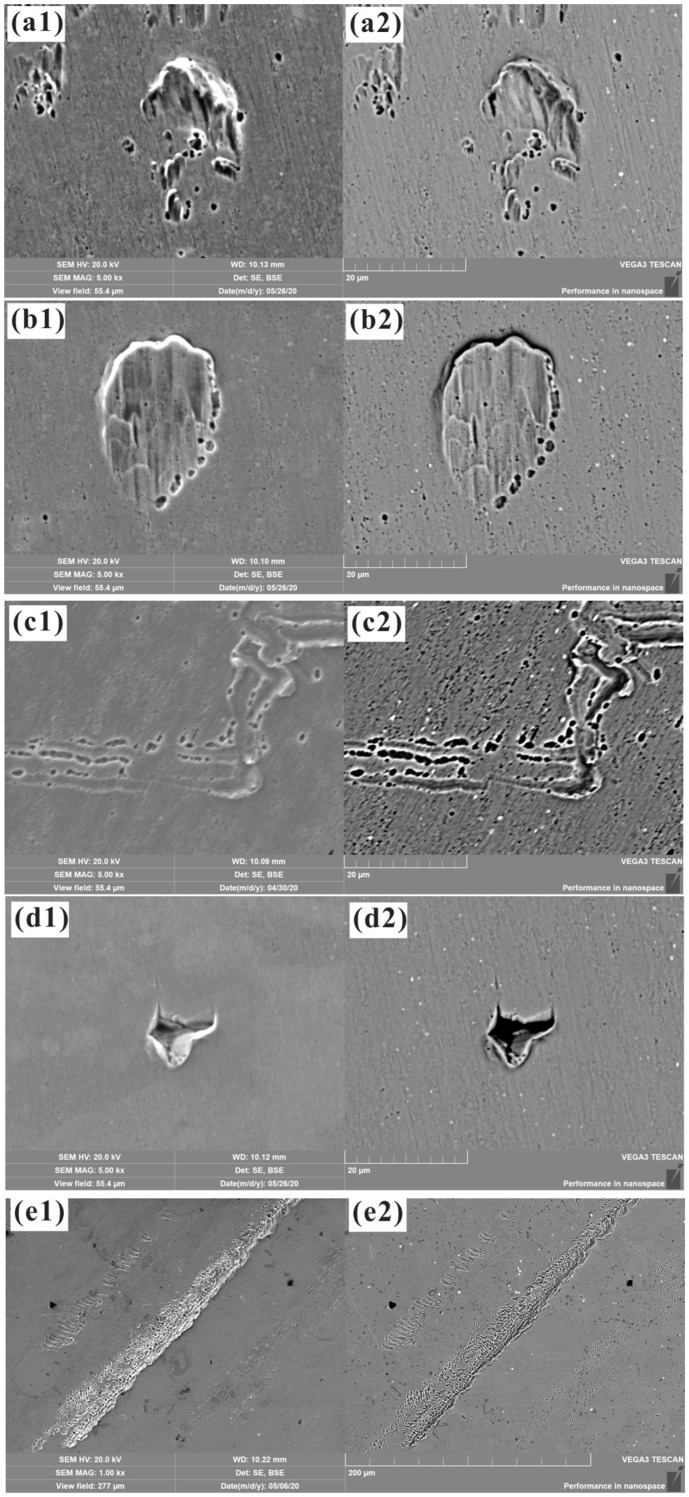
SEM images of the typical deformations observed on the surface of heat-resistant steel ((**a1**–**d1**) SE mode and (**a2**–**d2**) BSE mode) and powder superalloy ((**e1**) SE mode and (**e2**) BSE mode) samples after isostatic pressing: (**a1**,**a2**) pit; (**b1**,**b2**) dent; (**c1**,**c2**) scratch-like surface damage; (**d1**,**d2**) nonmetallic inclusion and surface tearing; (**e1**,**e2**) surface tearing.

Figure 3 shows the microstructure, defects, and microstrain pattern induced by isostatic pressing on the surface of heat-resistant steel. Different types of deformation characteristics for mode II strain can be clearly observed. Figure 3(a1,a2) shows the morphology of the pits observed via SEM in the SE and BSE modes, respectively. It was found that isostatic pressing led to the formation of pits for both mode II-A and mode II-B strains. The large microstrain observed for mode II-B might have occurred due to different deformation mechanisms, since damage initiation occurred with the generation of mode II-A strain and different damage features connected with each other or propagated, leading to mode II-B strain, which included mode II-A strain. Figure 3(b1,b2) illustrates a clearer view of the microstrain resulting from both mode II-A and mode II-B strains. The pit with a large microstrain seems to have a similar morphology to that of dimpled-fracture surfaces [30]. Interestingly, a micropit with mode II-A strain could be observed near the boundary of the pit subjected to a large microstrain, which seemed to appear first, before the formation of a large microstrain, and contributed to the formation of a pit with a large microstrain. Figure 3(c1,c2) shows that a scratch-like deformation and “tongues” occurred intermittently or continuously, including both II-A and II-B strain modes. A similar microstrain phenomenon was also observed in the fracture morphology of steel. In some local magnified views, tearing of the material was observed due to the complex stress state. For example, Figure 3(d1,d2) shows the tearing in a microregion surface near a nonmetallic inclusion. Surface tearing and the large strain observed after isostatic pressing were due to the incompatibility of the physical properties, such as hardness and elastic modulus, of the matrix and the nonmetallic inclusion. Figure 3(e1,e2) shows the presence of tearing after the isostatic pressing process for superalloys. As for the formation of “tongues” was induced by a cleavage fracture in BCC metals, the similar phenomenon observed here for the G115 specimen surface was also induced by a cleavage fracture except for the plastic deformation phenomenon found in the present work [1,31].

**Figure 4:**
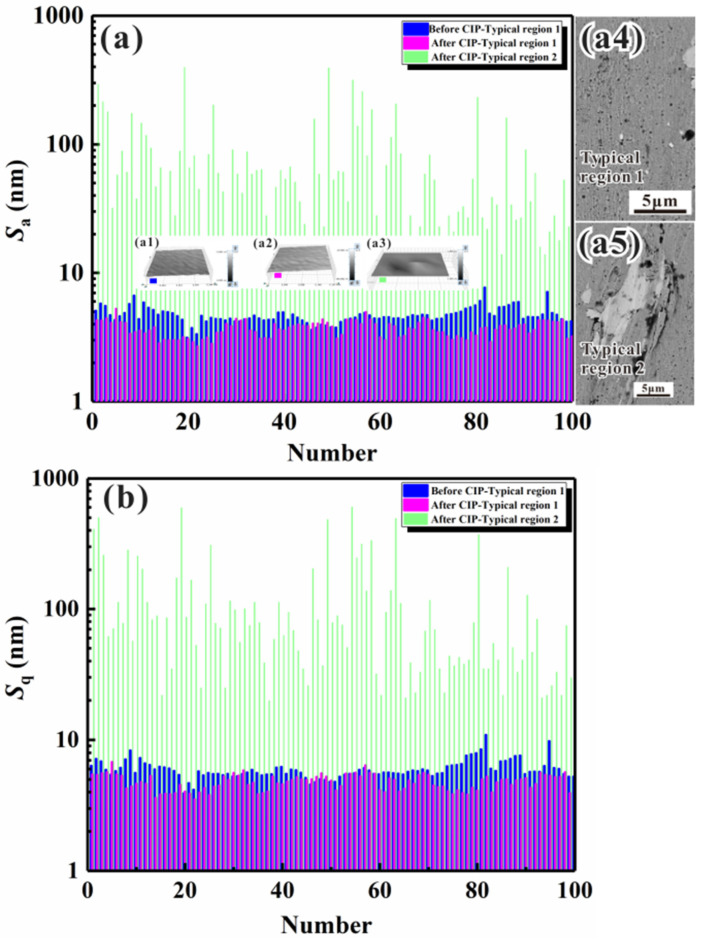
Mean vertical surface roughness (*S_a_*) and RMS vertical surface roughness (*S_q_*) for typical microregions with a small microstrain and collapse deformation generated on the surface of heat-resistant steel without the filtering process: (**a**) *S_a_*; (**a1**) 3D surface profile of a typical region 1 before isostatic pressing, (**a2**) 3D surface profile of a typical region 1 after isostatic pressing, (**a3**) 3D surface profile of a typical region 2 after isostatic pressing; (**a4**) SEM image of typical region 1; (**a5**) SEM image of typical region 2; (**b**) *S_q_*.

From the above deformation phenomena and strain modes, it is interesting to see that different microstrain mechanisms led to different types of microstrain behaviors. Therefore, to further classify the deformation behavior, it was important to first characterize the variation in surface profile and roughness after isostatic pressing. In the present work, the mean vertical roughness and root-mean-square (RMS) vertical roughness, denoted as *S_a_* and *S_q_*, respectively, were measured to obtain information on the surface vertical roughness [32,33]:

(1) $$S_{a}=\iint_{a}\left|Z\left(x,y\right)\right|dxdy$$

(2) $$S_{q}=\sqrt{\iint_{a}Z{\left(x,y\right)}^{2}dxdy}$$

where *Z*(*x*, *y*) represents the height of the surface profile, and *x* and *y* are the in-plane coordinates of the specimen. For the quantitative analysis of the vertical roughness variation, 100 different rectangular microregions with dimensions of about 100–300 × 100–300 μm^2^ were selected for the statistical measurement of *S_a_* and *S_q_*. As the length × width value was fixed, they could be compared for the specimens before and after CIP. Meanwhile, the samples before and after CIP were subjected to the same statistical analysis procedure to maintain consistency and comparability. To reduce shape errors, the samples were processed by Gaussian filtering in short-wave passing mode. A typical region I (low microstrain) was an area without pronounced variations in surface height, while a typical region II (large microstrain) was an area characterized by a large strain. The vertical roughness of the surface profile obtained before and after isostatic pressing (without any filtering process) can be seen in Figure 4, with Figure 4a,b presenting the statistical results of *S_a_* and *S_q_*, respectively. The insets in Figure 4a present the 3D surface profiles of local microregions in the center of the specimen obtained before (Figure 4(a1)) and after (Figure 4(a2,a3)) isostatic pressing. The results showed that the surface profile varied following two trends: increasing smoothness with decreasing surface vertical roughness (Figure 4(a1,a2)) and decreasing smoothness with increasing surface vertical roughness (Figure 4(a1–a3)). The statistical results also indicated that the surface vertical roughness of microregions with a low microstrain (region I) was far smaller than that of microregions with a large microstrain (region II). Moreover, by comparing these results with those obtained before isostatic pressing, it was found that both Sa and Sq were smaller for the smoothened microregions (region I without a pronounced surface height variation) in the specimen after isostatic pressing.

The above phenomena were likely due to the following main reasons: (1) the deformation was restricted within the level of a phase or of crystalline grains (low microstrain); (2) the deformation contained many phases or crystalline grains (collapse deformation). The decrease in surface vertical roughness after isostatic pressing could be induced by the plastic deformation of the constituent phases. From the surface profiles, the average value of the surface profile fluctuation (p) along the horizontal direction was calculated to be about 10–20 μm; this value was independent of the grain size but depended on the particle size of the diamond polishing fluid. We here considered a simple example with two constituent phases: a hard phase and a soft phase. After mechanical polishing, many peaks and valleys with a period of 10–20 μm formed due to the abrasion of the specimen surface by the diamond grit. Therefore, the peaks and valleys formed only hard phases, soft phases, or a combination of these two phases, depending on the relationship between the microstructure dimensions and the height fluctuation. As observed in Figure 2 and Figure 3, the microstructure dimensions were smaller than the fluctuation period, which indicated that each peak or valley contained many constituent phases. Once the liquid load was applied to the specimen surface, plastic deformation occurred, and the surface roughness decreased in many microregions. During isostatic pressing, the surface vertical roughness of protruding microregions decreased because of the plastic deformation due to the slip mechanism. According to a study by Dai et al. [34], there is a linear relationship between surface vertical roughness and plastic deformation and average grain size. As a result, in most surface areas, the surface height fluctuation and the surface vertical roughness decreased simultaneously. Under normal conditions, a homogeneous distribution of the microstructure and mechanical properties was considered in conventional studies of the deformation and mechanical properties of materials. However, due to the intrinsic inhomogeneity of the composition, microstructure, and defects, the mechanical properties of specimens may exhibit inhomogeneous characteristics [35]. Furthermore, the micromechanical properties of numerous microregions are statistically distributed due to the inhomogeneity of the composition, microstructure, and defects [36,37]. Under isostatic pressing, collapse deformation and damage initiation and propagation preferentially occur in microregions with poor micromechanical properties, such as areas that contain microcracks or subsurface pores (such as those that lead to a local depression during HIP [38]). The green solid lines in Figure 4 represent the surface vertical roughness of microregions that underwent collapse deformation at different strain degrees, ranging from tens of nanometers to microns. As mentioned above, the II-B strain mode represents a type of collapse deformation that might be induced by one microstrain mechanism or by several microstrain mechanisms (i.e., a combinatorial microstrain mechanism).

**Figure 5:**
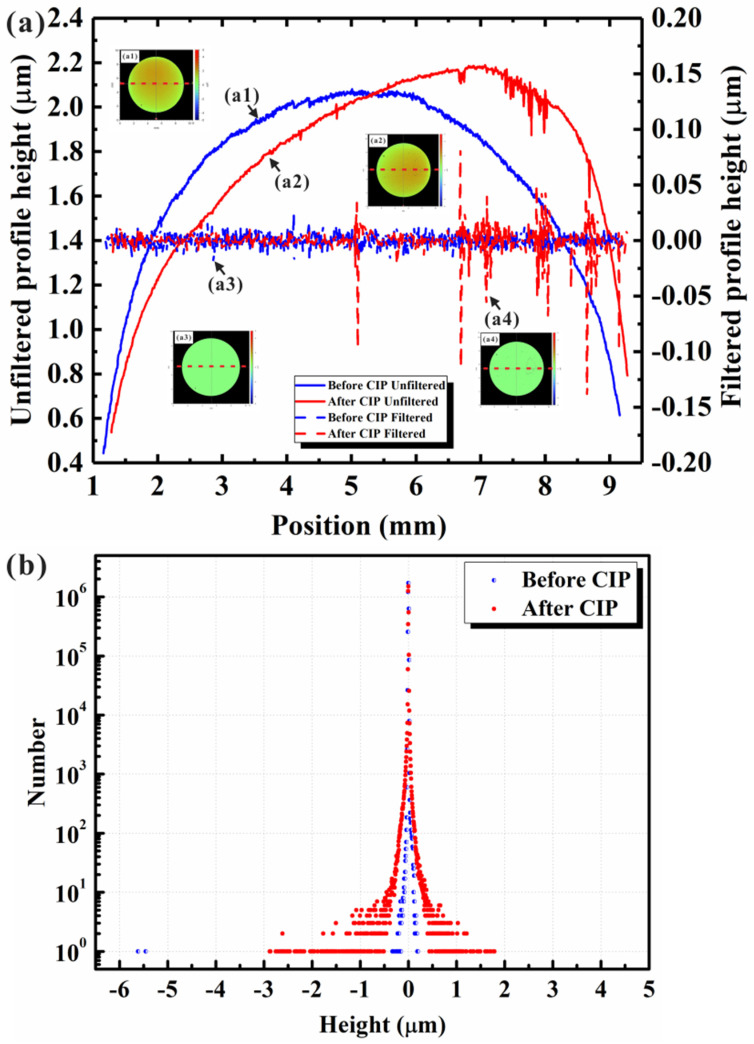
Original surface profiles for the selected circular regions with the same diameter obtained before and after isostatic pressing and corresponding data obtained after the filtering process: (**a**) original surface profiles and corresponding relative height information after filtering, (**a1**) before isostatic pressing and with no filtering, (**a2**) after isostatic pressing and with no filtering, (**a3**) before isostatic pressing, after filtering, (**a4**) after isostatic pressing and filtering; (**b**) relative height information after the filtering process for heat-resistant steel obtained before and after isostatic pressing.

Due to the small area of each measured region (areas with a low microstrain had dimensions of about 100–300 × 100–300 μm^2^), the selected regions without significant height fluctuations could be considered as having almost a flat surface, and the macroprofiles of the selected regions could be neglected, since the influence of the macroscopic shape on the surface vertical roughness was very small. However, the original surface profile of the specimen exhibited a height variation in the range of several microns, as shown in Figure 1a,b. Therefore, when evaluating the surface vertical roughness variation of the whole specimen area, the influence of the macroscopic shape on the surface vertical roughness could not be neglected. Surface data include information on both the macroscopic shape and the microscopic profile of a specimen surface and can be decomposed into two components: (1) low-frequency component (macroscopic profile); (2) high-frequency component (microscopic profile) [34]. In order to directly compare the surface profile data obtained before and after isostatic pressing, a filtering process of the macroscopic shape is required. In the present work, the Gaussian regression filtering process was used with a short wavelength cutoff to exclude the influence of the macroscopic shape on the surface vertical roughness and include the influence of the microstructure dimensions on the surface vertical roughness, since the microstructure dimensions were generally smaller than 100 μm [39,40]. Figure 5a shows the surface profiles obtained before and after isostatic pressing (red and blue solid lines, respectively) and the corresponding data after the Gaussian filtering process (red and blue dash lines, respectively). The insets (Figure 5(a1–a4)) in Figure 5a display the selected statistical microregions, and the results shown in Figure 5a were obtained from the red dash lines marked in Figure 5(a1–a4). The results shown in Figure 5a indicated that the surface profiles with no filtering could not be directly compared because of the simultaneous variation in the macroscopic shape. After the Gaussian filtering process with a zero-mean value, the corresponding surface data could be directly compared by evaluating the statistical distribution characteristics of the relative height data. The relative height data (after the Gaussian filtering process) shown in Figure 5 also indicated that the fluctuation in surface height decreased in most selected microregions but increased in some special microregions, which was in good agreement with the trend before the filtering process.

**Figure 6:**
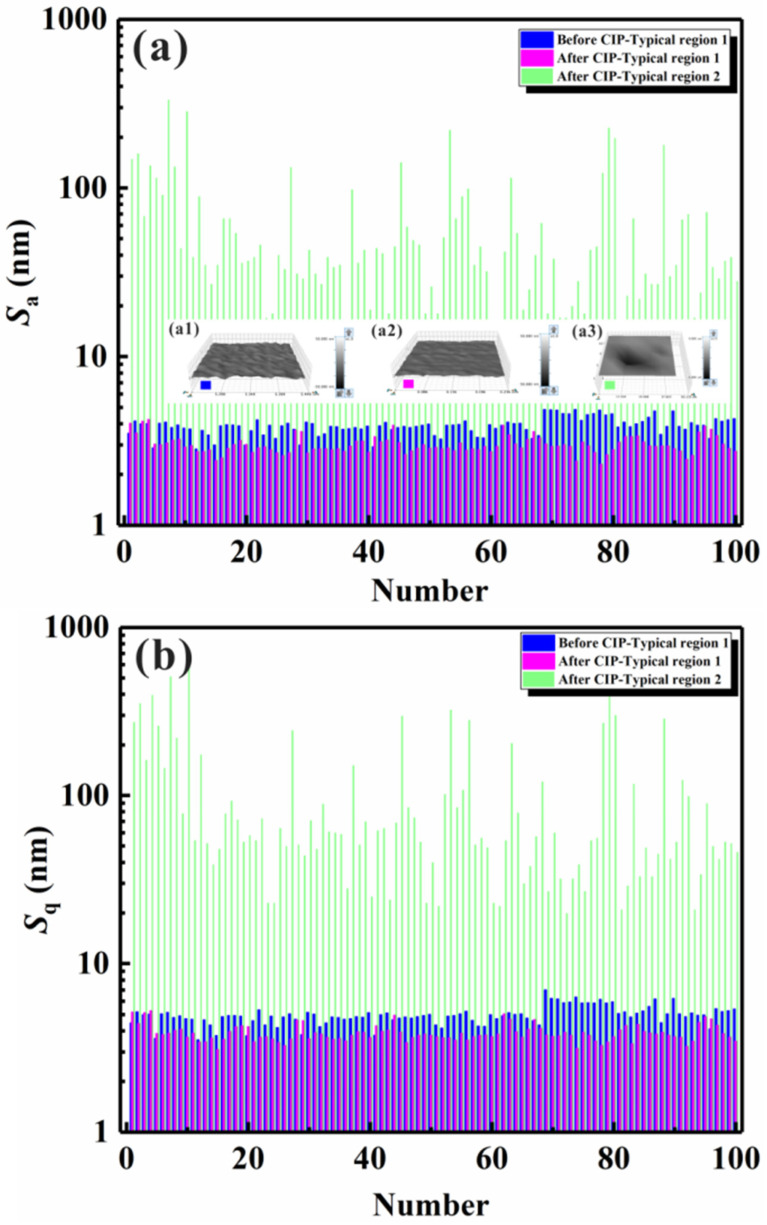
Surface vertical roughness of heat-resistant steel obtained after Gaussian filtering for two typical regions exhibiting a low microstrain and collapse deformation: (**a**) *S_a_*, (**a1**) 3D surface profile of a typical region 1 obtained before isostatic pressing, (**a2**) 3D surface profile of a typical region 1 obtained after isostatic pressing, (**a3**) 3D surface profile of a typical region 2 obtained after isostatic pressing; (**b**) *S_q_*.

Additionally, the corresponding surface vertical roughness values measured in different selected microregions after the filtering process were also compared, as shown in Figure 6. The insets in Figure 6a illustrate typical deformation microregions before (Figure 6(a1)) and after (Figure 6(a2,a3)) isostatic pressing. The height range scales in Figure 6(a1,a2) are the same as in the previous figure. The statistical results of the surface vertical roughness after filtering showed the same microstrain trend. After isostatic pressing, two types of microstrain trends could be detected, namely, low microstrains (corresponding to a decrease in surface vertical roughness) and large microstrains (corresponding to an increase in surface vertical roughness).

**Figure 7:**
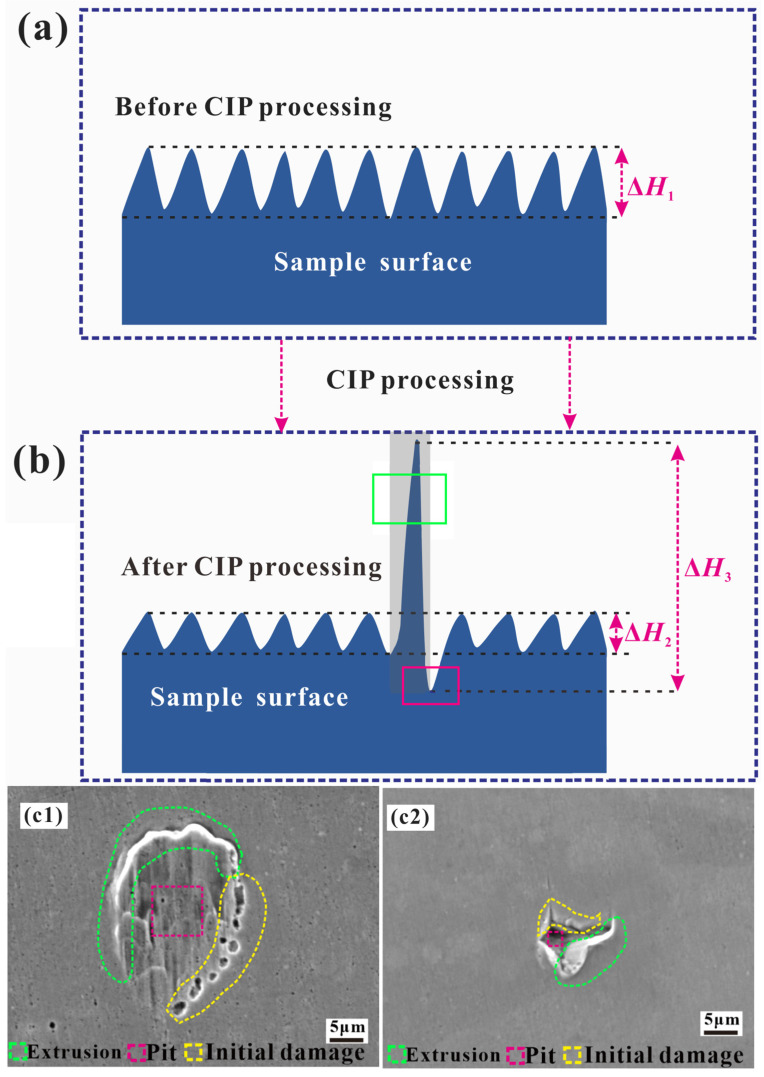
Schematic of the main deformation process of heat-resistant steel after isostatic pressing: (**a**) before CIP; (**b**) after CIP; (**c1,c2**) detailed deformation information after CIP.

As mentioned above, different types of microstrain behaviors were observed during the isostatic pressing process. However, for a more detailed analysis, a wider distribution of strain should be considered. Figure 7 shows the classification of the main observed microstrain phenomena on the surface of heat-resistant steel. On the basis of microstructure observation and surface profile analysis, the microstrain could be broadly classified according to two trends: (1) surface smoothening with decreasing height fluctuation in local microregions (ΔH1→ΔH2); (2) surface roughening with increasing height fluctuation in local microregions (ΔH1→ΔH3). Typically, the vertical roughness and horizontal roughness of a surface profile are independent, and a linear relationship was found between the magnitude of plastic deformation and the surface vertical roughness, as reported by Dai et al. [34]. Then, information on the height fluctuation provides information on the variation in surface vertical roughness. When the influence of the macroscopic shape on the surface vertical roughness can be neglected after the filtering process, the microstrain trend can also be described as (i) surface smoothening with decreasing surface vertical roughness and (ii) surface roughening with increasing surface vertical roughness. This roughening process is similar to the roughening phenomenon observed during plastic deformation, such as during the plastic straining of Al–Mg–Si sheets or austenitic stainless steels, although different processing techniques are used, and different principles are at the basis of these processes [41,42]. Furthermore, an increase in surface vertical roughness is also induced by several types of collapse deformation with different strain degrees. For example, when the deformation occurs in a restricted small microregion including one constituent phase or several constituent phases, the strain degree is very small, and the surface vertical roughness is only larger than the vertical roughness measured before isostatic pressing. When the deformation occurs in microregions containing many constituent phases, the strain degree increases. When the deformation occurs in regions with dimensions of hundreds of microns, the strain degree increases even more (for example, the surface vertical roughness reaches the micron scale). The collapse microstrain with increasing surface vertical roughness indicates damage initiation and propagation in local microregions with poor micromechanical properties, and evaluating the strain degree represents a new promising method for the high-throughput screening of microregions with poor micromechanical properties and the characterization of the micromechanical properties of materials.

**Figure 8:**
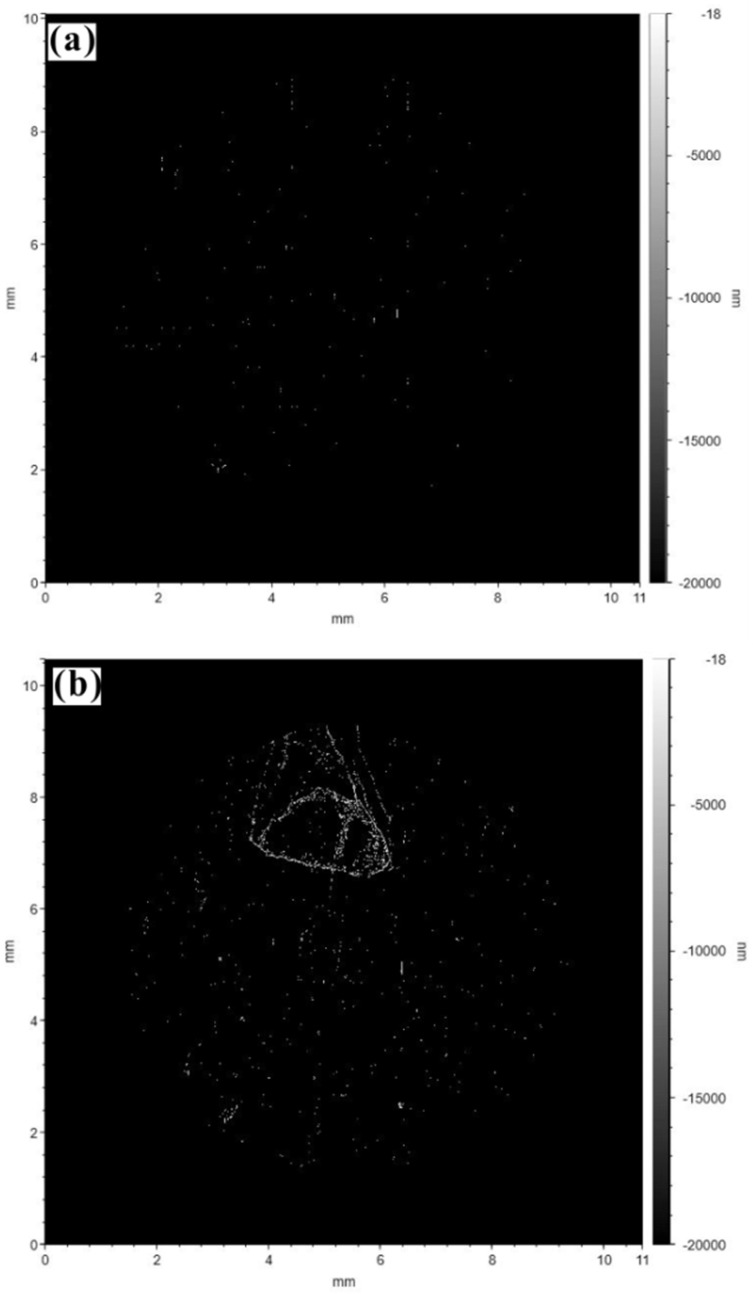
High-throughput screening and characterization of microregions on the surface of heat-resistant steel with values of the relative surface height: (**a**) before CIP; (**b**) after CIP.

**Figure 9:**
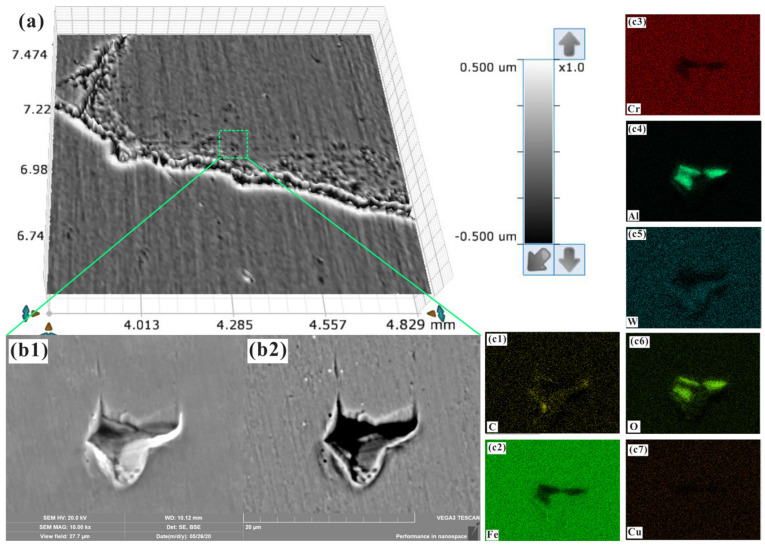
Scanning and location of the collapse deformation and the corresponding observed microstructures and analysis of the microregion composition: (**a**) location of the microregion with collapse deformation; (**b1**,**b2**) SE- and BSE-mode SEM images of the collapse deformation area; (**c1**–**c7**) EDS analysis of the collapse deformation area.

Figure 8 shows the statistical results of the relative height data after the Gaussian filtering process. The presence of microregions with significant height fluctuations could be observed directly from the experimental results; this can be considered a high-throughput screening method for studying the micromechanical properties or damage mechanics (microregions with high strain degrees and poor micromechanical properties, which might lead to failure during service) of materials. Similar to the high-throughput characterization of the composition, microstructure, or defects (such as nonmetallic inclusions) of materials, different strain degrees can be located, screened, statistically analyzed, and classified by setting different ranges of relative height [35,43,44,45]. Figure 9 shows the screening of microregions with poor micromechanical properties and a high strain degree. The collapse microstrain of microregions can be preliminarily detected on the basis of the surface profile information after isostatic pressing. From the SEM and EDS analyses, it can be concluded that the measured, large microstrain was induced by the tearing of the specimen surface around nonmetallic inclusions [46]. Similarly, based on the characterization [47,48,49] of the surface profile, composition, microstructure, and defects, as well as the related theoretical analysis of the deformation, the deformation mechanisms, such as inclusion tearing, plastic deformation, cleavage fracture, grain rotation, dislocation slip, or multiple combined deformation mechanisms, could be further screened and classified [50,51]. Combining isostatic pressing with the characterization of the surface composition, microstructure, defects, and deformation and depending on the type of relationship between composition, microstructure, defects, and strain, a new approach could be developed for the high-throughput screening and characterization of the micromechanical properties and damage mechanics of materials.

## 4. Conclusions

G115 heat-resistant steel was processed using the CIP technique, and then its composition, microstructure, and surface profile were characterized to clarify the effect of isostatic pressing on strain degree and surface deformation, as well as the possible applications of the technique used. After isostatic pressing, different types of deformation were observed on the surface of the G115 specimen via contour OM. Furthermore, the generation of surface deformation was observed, which was classified into two broad categories: low microstrain and large microstrain. The analysis of the surface vertical roughness showed the trend of microstrain modification after isostatic pressing: surface smoothening with decreasing surface vertical roughness (*S_q_*: 6.023 nm→4.799 nm) and surface roughening with increasing surface vertical roughness (*S_q_*: 6.023 nm→113.999 nm). The smoothening process of the surface was probably induced by plastic deformation, but the roughening process might have been induced by different mechanisms, such as cleavage fracture, tearing near nonmetallic inclusions, or plastic deformation. After the filtering process of the surface profiles, the corresponding relative height data could be directly compared, and the large microstrain could be screened and statistically characterized using a high-throughput method. In general, the analysis of the generation of collapse microstrain corresponding to a high strain degree in microregions with poor micromechanical properties and the high-throughput screening and statistical characterization of the micromechanical properties of the material could be achieved by simultaneously analyzing its composition, microstructure and defects, as well as the statistical distribution of the deformation on its surface.

## Data Availability

Data are contained within the article.
